# Comparative vector competence of the Afrotropical soft tick *Ornithodoros moubata* and Palearctic species, *O*. *erraticus* and *O*. *verrucosus*, for African swine fever virus strains circulating in Eurasia

**DOI:** 10.1371/journal.pone.0225657

**Published:** 2019-11-27

**Authors:** Rémi Pereira de Oliveira, Evelyne Hutet, Frédéric Paboeuf, Maxime Duhayon, Fernando Boinas, Adalberto Perez de Leon, Serhii Filatov, Laurence Vial, Marie-Frédérique Le Potier

**Affiliations:** 1 Swine Virology and Immunology Unit, Laboratoire de Ploufragan-Plouzané-Niort, Agence Nationale de Sécurité Sanitaire (ANSES), Ploufragan, France; 2 UMR ASTRE Animal Santé, Territoires, Risques et Ecosystèmes, Centre de coopération Internationale en Recherche Agronomique pour le Développement (CIRAD), Montpellier, France; 3 University of Montpellier, Montpellier, France; 4 CIISA—Centro de Investigação Interdisciplinar em Sanidade Animal, Faculdade de Medicina Veterinária, Universidade de Lisboa, Lisboa, Portugal; 5 Knipling-Bushland U.S. Livestock Insects Research Laboratory and Veterinary Pest Genomics Center, USDA-ARS, Kerrville, Texas, United States of America; 6 National Scientific Center Institute of Experimental and Clinical Veterinary Medicine, NSC IECVM), Kharkiv, Ukraine; Plum Island Animal Disease Center, UNITED STATES

## Abstract

African swine fever (ASF) is a lethal hemorrhagic disease in domestic pigs and wild suids caused by African swine fever virus (ASFV), which threatens the swine industry globally. In its native African enzootic foci, ASFV is naturally circulating between soft ticks of the genus *Ornithodoros*, especially in the *O*. *moubata* group, and wild reservoir suids, such as warthogs (*Phacochoerus* spp.) that are bitten by infected soft ticks inhabiting their burrows. While the ability of some Afrotropical soft ticks to transmit and maintain ASFV is well established, the vector status of Palearctic soft tick species for ASFV strains currently circulating in Eurasia remains largely unknown. For example, the Iberian soft tick *O*. *erraticus* is a known vector and reservoir of ASFV, but its ability to transmit different ASFV strains has not been assessed since ASF re-emerged in Europe in 2007. Little is known about vector competence for ASFV in other species, such as *O*. *verrucosus*, which occurs in southern parts of Eastern Europe, including Ukraine and parts of Russia, and in the Caucasus. Therefore, we conducted transmission trials with two Palearctic soft tick species, *O*. *erraticus* and *O*. *verrucosus*, and the Afrotropical species *O*. *moubata*. We tested the ability of ticks to transmit virulent ASFV strains, including one of direct African origin (Liv13/33), and three from Eurasia that had been involved in previous (OurT88/1), and the current epizooties (Georgia2007/1 and Ukr12/Zapo). Our experimental results showed that *O*. *moubata* was able to transmit the African and Eurasian ASFV strains, whereas *O*. *erraticus* and *O*. *verrucosus* failed to transmit the Eurasian ASFV strains. However, naïve pigs showed clinical signs of ASF when inoculated with homogenates of crushed *O*. *erraticus* and *O*. *verrucosus* ticks that fed on viraemic pigs, which proved the infectiousness of ASFV contained in the ticks. These results documented that *O*. *erraticus* and *O*. *verrucosus* are unlikely to be capable vectors of ASFV strains currently circulating in Eurasia. Additionally, the persistence of infection in soft ticks for several months reaffirms that the infectious status of a given tick species is only part of the data required to assess its vector competence for ASFV.

## Introduction

African Swine Fever (ASF) is a lethal disease of domestic pigs (*Sus scrofa domesticus*) and wild boar (*Sus scrofa scrofa*) caused by African swine fever virus (ASFV), which is enzootic in many countries of Sub-Saharan Africa. In the late 1950’s, due to increasing international trade, ASF spread out of the African continent, where ASFV is tick-borne in its natural cycle, and for the first time made inroads into Western Europe, South America and the Caribbean until the 1970’s. ASF was successfully eradicated from those regions through intense zoosanitary efforts, in some cases taking decades for completion, with the exception of the island of Sardinia [1]. However, ASF re-emerged during the 1990’s in the Iberian Peninsula where the native soft tick *O*. *erraticus* was identified as a natural reservoir of ASFV, allowing its long-term persistence and occasional re-emergence; several years were necessary to finally eradicate ASF from this region [2]. In 2007 ASF emerged in Eurasia, first in Georgia [3] and then spread through the Russian Federation to Eastern and Central Europe westwards eventually affecting pigs in country members of the European Union in 2014 [4]. By 2018, ASFV was detected in Belgium [5], meanwhile ASF affected pigs in farther parts of Asia for the first time, including China, to then spread further in this continent [6]. There is no vaccine neither treatment available to control ASF in domestic pigs and wild boar [7, 8]. Quarantine, herd depopulation, and zoning are practiced for the eradication of ASF outbreaks and to prevent the spread of ASF [9].

ASFV is an enveloped double-stranded DNA and soft tick-borne virus, and the only member of the *Asfarviridae* family. There are 24 ASFV genotypes currently known, some of which exhibit high diversity in Eastern and Southern Africa that may be the result of the existence of complex sylvatic and domestic transmission cycles in those regions involving wild and domestic *Suidae* as well as soft tick species of the *Ornithodoros* genus [10]. Transmission of ASFV to susceptible swine can occur via multiple routes including direct contact with infected suids, contact with contaminated carcasses or fomites, ingestion of contaminated food, and through the bite of infected soft tick vectors [11].

In native African areas where sylvatic transmission of ASFV occurs, soft ticks of the *Ornithodoros moubata* group are considered the main vectors and reservoirs for ASFV [12–14]. Previous experimental studies showed that some of these species can be orally infected, maintain and transmit the virus vertically (transstadially and transovarially) among ticks, and horizontally to naïve pigs. However, the success of transmission in the laboratory was shown to differ from one tick/virus combination to the other and to be influenced by the experimental design applied to infect the ticks [15–21]. In Western Europe, *O*. *erraticus* was shown to transmit some ASFV strains under field conditions [22, 23] and in laboratory experiments [24]. Furthermore, ASFV was isolated from *O*. *erraticus* that were collected at field sites where outbreaks had occurred more than five years prior, and successful experimental transmission to pigs was achieved with tick batches tested up to 380 days after an outbreak, which confirmed the importance of this soft tick species as an ASFV reservoir [2]. Experiments also demonstrated the susceptibility of *O*. *erraticus* to infection using ASFV strains classified in genotype I (Tomar/87, OurT88/1, ASFV/P99, NH/P68) [25–27]. This soft tick species was shown to remain infected with Georgia2007/1 from genotype II for a few weeks but its ability to transmit the virus has not been yet explored [28].

The current spread of ASF across Europe and Asia raises the critical question of how ASFV persists in the environment. ASFV transmission to naïve pigs can occur in a contaminated environment through exposure to excretions from infected pigs [29]. Pigs may become infected by ingesting biting flies like the stable fly, *Stomoxys calcitrans*, carrying ASFV [30]. Studies on the soft tick fauna in the Paleartic region identified species that could be ASFV vectors [31–33]. However, limited information exists on the vector competence of Palearctic soft tick species for ASFV strains currently circulating in Eurasia [34, 35]. The potential involvement of Palearctic soft ticks as ASFV vectors is further complicated by the fact that Georgia2007/1, which is the ASFV strain introduced from Eastern Africa into the Caucasus region in 2007 [3], seems to have evolved into genetically divergent and/or less virulent strains depending on the Eurasian area under consideration [36–39].

Here, we report the results of comparative experiments that tested the vector competence of the Palearctic soft tick species *O*. *erraticus* and *O*. *verrucosus*, and the Afrotropical species *O*. *moubata* sensu stricto (s.s.) for ASFV strains isolated in Eurasia, some of which are causing outbreaks in this region. All the soft tick species tested were susceptible to infection, but only *O*. *moubata* transmitted ASFV to pigs under our experimental conditions. Naïve pigs showed clinical signs of ASF when inoculated with homogenates of crushed *O*. *erraticus* and *O*. *verrucosus* ticks that fed on viraemic pigs, which proved the infectiousness of ASFV contained in the ticks. These results are discussed in the context of the epidemiology of ASF in Eurasia and other data on vector competence of Palearctic soft tick species for ASFV.

## Materials and methods

### ASFV strains and cells

Four different highly virulent ASFV strains were used for this study. Two of them belong to genotype II: Georgia 2007/1 ASFV strain, initially isolated in 2007 from a domestic pig originating in Georgia [3] that was kindly provided by Dr. Linda Dixon (OIE reference laboratory, Pirbright Institute, UK); and Ukr12/Zapo strain [40], isolated in 2012 from a domestic pig in Ukraine that was kindly provided by Dr Carmina Gallardo (ASF European Union Reference Laboratory,CISA-INIA, Valdeolmos, Spain). Both strains are circulating in Eurasia [39, 40]. The two other strains kindly provided by Dr. Linda Dixon (OIE reference laboratory, Pirbright Institute, UK) belong to genotype I: Liv13/33 isolated in Zambia in 1983 from *O*. *moubata* [41], and OurT88/1 isolated in Portugal in 1988 from *O*. *erraticus* [42].

The four ASFV strains were cultured on porcine alveolar macrophages once (Liv13/33; Ukr12/Zapo) or twice (Georgia2007/1; OurT88/1) before being intramuscularly inoculated in pigs [43]. Viruses were diluted in RPMI medium to adjust the inoculation dose to 10^4^ hemadsorbing dose 50% (HAD_50_) per pig. Virus titration was performed by hemadsorption assay [44].

### Soft ticks

The *Ornithodoros* soft tick species used in this study were: (i) *O*. *moubata sensu stricto* from Southern Africa, “Neuchâtel” strain maintained in Neuchâtel University insectary for at least twenty years and reared in CIRAD Montpellier since 2008), (ii) *O*. *erraticus* from Alentejo in Portugal (“Alentejo” strain, collected from the field in 2013 and 2016 and reared in CIRAD Montpellier with 1–5 generations completed since 2016), and (iii) *O*. *verrucosus* from Ukraine (collected from the field in 2014–2015 and reared in NSC IECVM, Kharkiv, with only 1 generation completed). Soft ticks were maintained in the laboratory at 26°C with 80 to 90% relative humidity as recommended for these species [45]. Only late instar of nymphs, females and males were used for the study to assure maximum ingestion of blood and associated ASFV particles for tick infection.

### Pigs

Sixty-eight Specific Pathogen-Free (SPF) Large White pigs, 7–10 weeks-old, were used in the experiments described below. They were identified individually and randomly housed in the air-filtered biosafety level 3 animal facilities at Anses-Ploufragan.

All pigs were monitored daily for rectal temperature and clinical signs of ASFV infection (recumbency, skin hemorrhage, diarrhea, vomiting, joint edema, blood in urine, laborious breathing, ocular discharge, and lack of appetite assessed by weighing unconsumed food). Each clinical sign was scored on a scale from 0 to 5, as previously described [43]. At the end of the experiment, or at earlier stages for animal welfare reasons (clinical score ≥ 15/40), pigs were humanely euthanized by anesthetic (Zoletil® 100) overdose at 5 mL per 50 kilograms of weight administered via the vena cava and then exsanguinated. Days of euthanasia are detailed in Tables 2, 3, 4 and 5.

Once per week and just before euthanasia, pigs were weighed. Blood samples were collected before the first exposure to ASFV-infected ticks, on the first day of hyperthermia, and then twice a week. Blood was collected in tubes with lithium heparin for viral titration, EDTA for ASFV genome detection by real-time Polymerase Chain Reaction (PCR), and dry tubes to obtain serum.

### Ethics statement

Animal experiments were authorized by the French Ministry for Research (project N° 2017062615498464) and approved by the national ethics committee (authorization N° 11/07/17-3).

### Tick infection and ASFV transmission experiments

**Fig 1:**
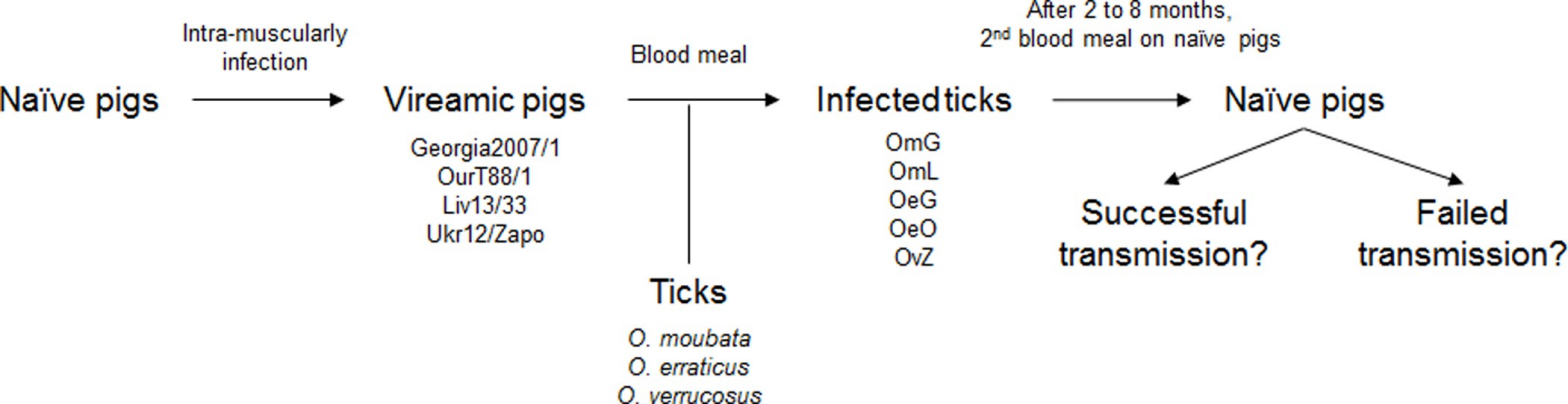
Experimental outline of experiments using viremic pigs to attempt soft tick infection depicting subsequent tests for ASFV transmission to pigs. Viremia was produced by inoculating naïve pigs intramuscularly with ASFV. Infection of three *Ornithodoros* soft tick species was attempted by allowing the ticks to blood feed on the viremic pigs. Two to eight months after feeding on viremic pigs, additional experiments were conducted to test the ability of these ticks to transmit different ASFV strains while acquiring a blood meal on naïve pigs. OmG: *O*. *moubata*-Georgia2007/1, OmL: *O*. *moubata*-Liv13/33, OeG: *O*. *erraticus*-Georgia2007/1, OeO: *O*. *erraticus*-OurT88/1, OvZ: *O*. *verrucosus*-Ukr12/Zapo.

Fig 1 depicts the series of experiments conducted to assess soft tick vector competence for ASFV. Briefly, pigs became viremic 3–4 days after intramuscular inoculation of the respective ASFV strain dose of 10^4^HAD_50_/pig. Ticks were fed on the pigs and then tested 2 to 8 months later for their ability to transmit ASFV to naïve pigs.

Table 1 summarizes the permutations tested in two trials attempting to infect ticks with ASFV feeding on viremic pigs. In the first trial, each of four groups of pigs was infected with one of the four ASFV strains listed above. When viremic, the pigs were used to blood feed cohorts of *O*. *moubata* s.s., *O*. *erraticus*, and *O*. *verrucosus*, which subsequently were used in vector competence tests. In the second infection trial, two groups of 3 pigs inoculated respectively with OurT88/1 or Georgia2007/1 were used to try to infect *O*. *erraticus* only. For both trials, a group of two healthy pigs was kept as control. The trials were conducted six months apart.

**Table 1:** Soft ticks fed on viremic pigs in two trials to attempt infection with four ASFV strains. Counts of engorged ticks for each *Ornithodoros* species tested are shown next to the number of pigs in brackets that were infected with the respective ASFV strain per trial.

	ASFV strain Ticks	Georgia2007/1	Ukr12/Zapo	Liv13/33	OurT88/1
1^st^ trial	*O*. *erraticus*	281 (4)			180 (3)
	*O*. *moubata*	313 (3)		260 (3)	
	*O*. *verrucosus*		145 (3)		
2^nd^ trial	*O*. *erraticus*	300 (3)			293 (3)

Ticks were fed on infected pigs during the first day of hyperthermia as previously described [46]. At this time, viremia in the pigs ranged from 10^7,5^HAD_50_/mL to 10^8,25^HAD50/mL. When ticks did not engorge during the initial blood meal, they were exposed again to the infected pigs the day after to attempt maximal tick infection. Thus, the five possible combinations of soft tick-ASFV infection included: *O*. *moubata-*Liv13/33 (OmL), *O*. *moubata*-Georgia2007/1 (OmG), *O*. *erraticus*-OurT88/1 (OeO), *O*. *erraticus*-Georgia2007/1 (OeG) and *O*. *verrucosus*-Ukr12/Zapo (OvZ).

### ASFV transmission by ticks feeding on naïve pigs.

Ticks representing the five possible infection pairs listed above were tested for their ability to transmit ASFV to naïve pigs (Fig 1). In each trial, the control group was not exposed to uninfected ticks because previous experiments did not show interference of the tick saliva with the ASFV transmission, pig infection and outcome of the disease in domestic pig [46].

#### 1) Transmission by *O*. *moubata*

At two months post infection (PI), OmL and OmG were allowed to feed on naïve pigs in a single tick challenge with 30 ticks per pig to compare the ability of this tick species to transmit two different ASFV strains. For both tick-virus pairs, two naïve pigs were exposed to tick feeding. To evaluate the reservoir abilities of both tick-virus pairs, one naïve pig was challenged with 36 OmL at 8 months PI, and two naïve pigs were challenged with 40 OmG at 6 months PI. In a subsequent experiment, two naïve pigs were challenged with 60 descendants of OmL per animal based on previous findings [19]. Nymphal ticks in the first stage (N1) obtained from eggs laid by ASFV infected females during the second gonotrophic cycle were used as the first cycle is apparently too close to the infective blood meal to allow transovarial passage of the virus. These experiments included one contact pig, which received no treatment but remained in close contact with the challenged pigs except the OmL at 8 months PI that had two contact pigs.

#### 2) Transmission by *O*. *erraticus* and *O*. *verrucosus*

For OeO, OeG, and OvZ at 2 months post infection, two naïve pigs were challenged once with 30 ticks per animal. Additionally, to test for a dose effect in ASFV transmission, two naïve pigs were challenged with: 1) 142 and 147 OeG at 2 months PI; and 2) 143 OeO at 2 months PI. Each of these experiments included one contact pig. To test the effect of time post- tick infection in *O*. *erraticus*, one naïve pig was challenged with 116 OeG at 8 months PI. This experiment included two contact pigs.

#### 3) Repeated exposure to *O*. *moubata* and *O*. *erraticus* infected with Georgia2007/1

A test for the effect of repeated exposure of pigs to bites by infected ticks was conducted. To achieve this, triple-challenge transmission trials were done using the OmG and OeG ticks at 2 months PI. The three challenges were spaced by 3–4 days each using a new batch of 15 ticks/pig/challenge. A contact pig was included for each of the two tick-virus pairs.

### ASFV transmission to naïve pigs by intramuscular inoculation of crushed-tick homogenates

This experiment was conducted following the lack of transmission observed after exposure of naïve pigs to infected ticks, namely OvZ at 2 months PI, OeO at 2 months PI, and OeG at 2 and 8 months PI. Ten ticks per tick-virus pair were crushed individually in 200μL of sterile phosphate buffered saline solution (PBS) using a Star-Beater (VWR) with one bead of 3 mm and one bead of 4 mm at 25 Hz during 3 mins. To each tube, 800μL of RPMI medium was added and the content was centrifuged at 2000g during 2 mins. For OeO and OeG at 2 months PI, 100μL of the supernatant from each individual tick was pooled into 1 mL of RPMI to a final volume of 2 mL and then inoculated to one naïve pig intramuscularly. For OvZ at 2 months PI and OeG at 8 months PI, 500μL of the supernatant from individual ticks was pooled to a final volume of 5 mL and then inoculated to one naïve pig. For the last two groups, 5 mL was inoculated because the ASFV DNA load, measured by real-time PCR and expressed by cycle threshold (Ct), was lower (26.04–39.83 Ct) than the other groups (20–29.86 Ct).

### ASFV detection and serological assay

Pigs were diagnosed for ASFV infection by real-time PCR as previously described [47] using DNA extracted from 100μL of EDTA blood samples using the DNeasy Blood and Tissue kit (Qiagen, Courtaboeuf, France). Pig Beta-actin was used as internal control for DNA extraction from pig samples. ASFV was also detected in ticks by real time PCR using primers and probes targeting ASFV VP72 as previously described [47]. To have an internal control, primers to amplify tick beta-actin [48] and the probe Hex-5’-CGAGAGGAAGTACTCCGTCTGG-3’-BHQ1 were added to each PCR mixture. Real time PCRs were performed on 200 μL of each crushed tick supernatant after DNA extraction using High Pure PCR Template Preparation Kit (Roche Life Science). Additionally, mosquito nets of the tick feeding units used for the transmission trials to pigs were washed in 5 mL of PBS to extract DNA using High Pure PCR Template Preparation Kit (Roche Life Science), which was used to perform the ASFV real-time PCR described above.

Antibodies against ASFV were measured in serum obtained from the last blood sample collected before the pigs were euthanized, which corresponded to less than one week for infected pigs and more than 16 days for the other pigs to explore possible seroconversion in groups where transmission by tick bite failed. Serum samples were purified by centrifugation at 3000g for 5 mins and anti-ASFV antibody detection was done using a competition ELISA kit per the manufacturer’s instructions (INGENASA PPA3 COMPAC, Spain).

### Statistical analysis

To test the effect of the route of pig infection on the evolution of the disease (clinical score and onset of hyperthermia), the following treatment groups were established: 1) pigs infected by intramuscular inoculation of ASFV, 2) pigs infected by tick feeding, and 3) pigs infected by inoculation of homogenates of crushed ticks.

Statistical analyses were performed using the RStudio software (version 1.1463). All analyses were run using generalized mixed effect models with pigs as random effect (to account for the non-independence of the data), whereas treatment and time were fitted as fixed effects (interaction between the two terms was not significant). The speed of hyperthermia onset was analyzed with a Poisson distribution whereas clinical score required a negative binomial distribution to account for over dispersion of the data.

## Results

### Confirmation of tick infection and feeding on naïve pigs to test ASFV transmission

**Fig 2:**
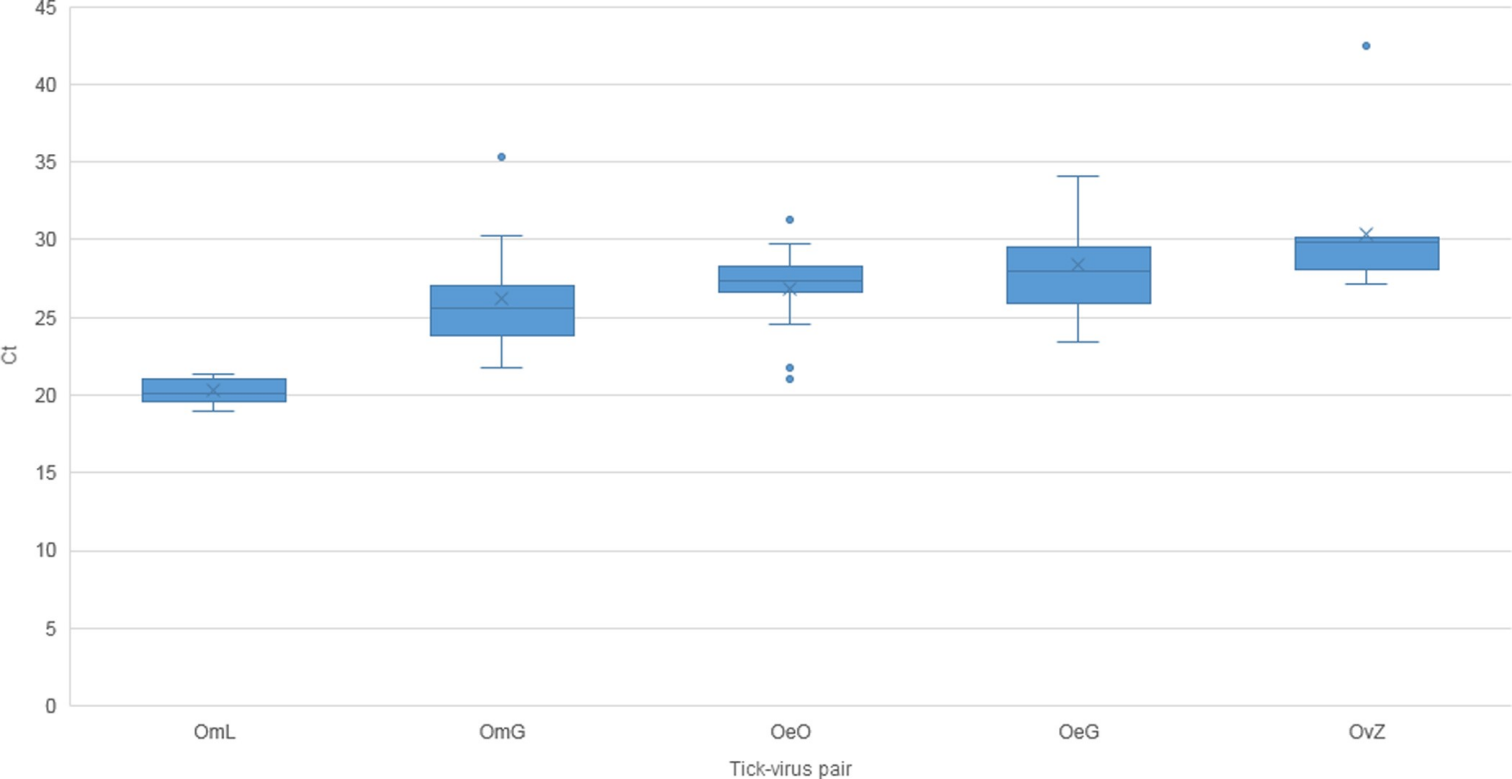
Detection of ASFV VP72 gene in soft ticks by real time PCR. ASFV infection was tested in soft ticks for the following viral strain associations: *O*. *moubata*-Liv13/33 (OmL), *O*. *moubata*-Georgia2007/1 (OmG), *O*. *erraticus*-OurT88/1 (OeO), *O*. *erraticus*-Georgia2007/1 (OeG) and *O*. *verrucosus*-Urk12/Zapo (OvZ). Ct = Cycle Threshold.

Tick infection was assessed by real-time PCR two months PI and before the transmission trials started. Fifteen ticks for each of the OmL, OmG, OeO, and OeG virus pairs, and 10 ticks for the OvZ pair were tested. All the ticks tested were PCR-positive for ASFV (Fig 2).

Following blood feeding on naïve pigs for transmission trials, ticks were sorted by life stage (nymph or adult) and sex of the adults (male or female), and divided according to feeding status into “engorged” vs “non-engorged” groups. Engorgement rates in the two trials ranged from 68.8 to 100% depending on the tick-virus pair tested (Tables 2 and 3). ASFV was detected by PCR (Ct: 32.86–40.46) in liquid used to wash mosquito nets in the feeding units containing tick excreta (coxal fluid and feces) for all the tick-virus pairs.

**Table 2:** ASFV transmission by *O*. *moubata* ticks. Experiments involved ticks previously exposed to ASFV strains OmL, OmG, and the descendants of OmL ticks. F = female, M = male, N = nymph, OmL = *O*. *moubata*-Liv13/33, OmG = *O*. *moubata*-Georgia2007/1.

Pig identification	Tick-virus pair	Time post tick infection	Ticks fed: engorged/total (% engorgement)	Sex of engorged tick	First day of hyperthermia	Day of euthanasia	ASFV diagnosis
SwineML1	OmL	2 months	29/30 (96.6)	11F/18M	2	3	+
SwineML2			27/30 (90)	9F/18M	2	3	+
SwineML3		8 months	27/36 (75)	17F/10M	2	4	+
SwineMG1	OmG	2 months	28/30 (93.3)	12F/15M/1N	3	4	+
SwineMG2			29/30 (96.6)	16F/12M/1N	4	4	+
SwineMG3		6 months	39/40 (97.5)	22F/17M	3	5	+
SwineMG4			39/40 (97.5)	21F/18M	3	5	+
SwineDML1	OmL descendants	3 months	47/60 (78.3)	47N	3	5	+
SwineDML2			42/61 (68.8)	42N	2	5	+

**Table 3:** Lack of ASFV transmission by *O*. *erraticus* and *O*. *verrucosus* ticks. Experiments involved ticks previously exposed to ASFV strains. F = female, M = male, N = Nymph, OeO = *O*. *erraticus*-OurT88/1, OeG = *O*. *erraticus*-Georgia2007/1, OvZ = *O*. *verrucosus*-Zapo.

Pig identification	Tick-virus pair	Time post tick infection	Ticks fed: engorged/total (% engorgement)	Sex of engorged tick	Day of euthanasia	ASFV diagnosis
SwineEO1	OeO	2 months	29/30 (96.6)	23F/4M/2N	28	-
SwineEO2			27/30 (90)	20F/6M/1N	28	-
SwineEO3			140/143 (97.9)	51F/48M/41N	20	-
SwineEO4			132/143 (92.3)	50F/44M/38N	20	-
SwineEG1	OeG	2 months	29/30 (96.6)	20F/8M/1N	27	-
SwineEG2			25/30 (83.3)	21F/4M	27	-
SwineEG3			138/147 (93.4)	42F/42M/44N	20	-
SwineEG4			133/142 (93.6)	43F/51M/39N	20	-
SwineEG5		8 months	107/116 (92.2)	86F/18M/3N	16	-
SwineVZ1	OvZ	2 months	24/30 (80)	6F/10M/8N	29	-
SwineVZ2			27/30 (90)	9F/1M/17N	29	-

### *O*. *moubata* transmission of the ASFV Georgia2007/1 and Liv13/33 strains

Two months PI, ticks representing the OmL and OmG associations transmitted ASFV to naïve pigs (SwineML1/SwineML2 and SwineMG1/SwineMG2, respectively). The two pigs bitten by OmL ticks were hyperthermic on day 2 after tick feeding while the two other pigs exposed to OmG ticks were hyperthermic on day 3 and day 4, respectively, after the ticks blood fed on them (Table 2). Eight and 6 months PI, respectively, OmL and OmG ticks were still able to transmit ASFV to naïve pigs (SwineML3 and SwineMG3/SwineMG4). The pig bitten by OmL ticks was hyperthermic on day 2 after the exposure while the two other pigs bitten by OmG ticks were hyperthermic on day 3 (Table 2). Pigs SwineDML1 and SwineDML2 exposed to descendants of OmL ticks were hyperthermic on day 2 and 3, respectively, post tick exposure. All pigs exposed to OmL and OmG ticks were ASFV PCR positive (Table 2). The contact pigs remained PCR-negative.

### Lack of ASFV transmission by *O*. *erraticus* and *O*. *verrucosus*

Pigs bitten by 30 ticks of the OeO, OeG or OvZ pairs 2 months PI remained alive during a period of 23 days and without any ASF clinical signs or viraemia (Table 3). No antibodies against ASFV were detectable by ELISA in these pigs at day 23 after tick feeding.

### ASFV in *O*. *erraticus* and *O*. *verrucosus* was not transmitted to, but remained infective for naïve pigs

All naïve pigs inoculated with homogenates obtained from OeO, OeG and OvZ ticks became hyperthermic between 3 to 5 days post inoculation (Table 4). This outcome confirmed that ASFV contained in the ticks remained infectious. These pigs were ASFV positive by PCR (Table 4). The same results were observed when homogenates for the inoculation were obtained from OeG ticks that had fed on a viremic pig 8 months before their ability to transmit ASFV was tested.

**Table 4:** Clinical outcome in pigs inoculated with homogenate supernatant of *O*. *erraticus* and *O*. *verrucosus* previously exposed to selected ASFV strains. Experiments involved ticks previously exposed to ASFV strains. OeO = *O*. *erraticus*-OurT88/1, OeG = *O*. *erraticus*-Georgia2007/1, OvZ = *O*. *verrucosus*-Zapo.

Pig identification	Tick-virus pair	Time post tick infection	Volume of supernatant	First day of hyperthermia	Day of euthanasia	ASFV diagnosis
SwineIEO1	OeO	2 months	1 mL	3	5	+
SwineIEG1	OeG		1 mL	4	6	+
SwineIVZ1	OvZ		5 mL	5	6	+
SwineIEG2	OeG	8 months	5 mL	3	5	+

### Lack of ASFV transmission after repeated blood feeding by OeG and OmG ticks

Pigs exposed to blood feeding three successive times by 15 ticks/pig/exposure with the OeG or OmG ticks at 2 months PI remained alive without showing clinical signs during the 30 days post-exposure period (Table 5). These pigs were also negative for ASFV by PCR and ELISA.

**Table 5:** Failure of ASFV transmission after repeated blood feeding by *O*. *erraticus* and *O*. *moubata* ticks previously exposed to selected ASFV strains. Proportions of soft ticks fed and their life stage and sex per experiment are shown. F = Female, M = male, N = Nymph. OeG = *O*. *erraticus*-Georgia2007/1, OmG = *O*. *moubata*-Georgia2007/1.

Pig identification	Tick-virus pair	Time post tick infection	Ticks fed: engorged/total, (% engorgement)	Life stage and sex of engorged ticks	ASFV diagnosis	Day of euthanasia
SwineEG6	OeG	2 months	14/15 (93.3), 15/15 (100), 15/15 (100)	9F/3M/2N, 9F/5M/1N, 12F/1M/2N	-	30
SwineEG7			15/15 (100) 15/15 (100), 15/15 (100)	7F/7M/1N, 12F/2M/1N, 10F/4M/1N	-	30
SwineMG5	OmG		13/15 (86,67), 15/15 (100), 15/15 (100)	8F/5M, 7F/7M/1N, 7F/8M	-	30
SwineMG6			15/15 (100), 15/15 (100), 15/15 (100)	10F/5M, 7F/8M, 7F/8M	-	30

### Clinical scores of infected pigs

Clinical scores of infected pigs with ASFV are shown in S1 Fig. No statistical difference was detected between pigs infected either by intra-muscular injection, tick bite, or inoculation of tick homogenate (Chisq = 3.725; p-value = 0.155). No statistical difference was found for the onset of hyperthermia between these three groups (Chisq = 1.090; p-value = 0.579). Control pigs had a clinical score of 0 during all the experiments.

## Discussion

To the best of our knowledge, this is the first study where vector competence for ASFV strains circulating in Eurasia was compared between Palearctic soft tick species, in this case *O*. *erraticus* and *O*. *verrucosus*, and the known Afrotropical soft tick vector *O*. *moubata*. The experimental design followed here closely mimicked natural conditions under which pathogen acquisition and transmission by biological soft tick vectors take place. Blood feeding on pigs facilitates engorgement of soft ticks, which is important for ASFV transmission but also for tick infection; pigs are much attractive than artificial membranes for promoting tick feeding and host infection with detectable viremia. Pigs were bitten by heterogeneous tick cohorts varying in numbers and consisting of different developmental stages and sexes. Keeping the pigs together to minimize stress when exposed to tick feeding prevented the use of chemical restraint. In addition to testing for virus uptake, the period elapsed between feedings also allowed infected tick to bite at different times PI, and single or multiple biting events by the same ticks as it occurs in the field. Thus, the approach taken with the experiments presented herein is biologically relevant as compared to laboratory vector competence studies where artificial feeding systems are used [49, 50]. Additionally, using negative controls receiving no treatment and positive control pigs that were infected by intramuscularly injection, which is the reference method to infect pigs experimentally with ASFV, afforded the measurement of clinical symptoms observed in pigs after they were bitten by infected ticks [43]. Pigs infected either by intramuscular injection or *Ornithodoros* tick bites displayed similar disease outcome with similar clinical score kinetics and a comparable onset of hyperthermia.

The Palearctic soft tick species *O*. *erraticus* and *O*. *verrucosus* were unable to transmit the ASFV strains tested to susceptible pigs under the experimental conditions of this present study. However, inoculation of naïve pigs with a homogenate of pooled infected ticks revealed that ASFV remained infectious during at least two months in OeO, OeG and OvZ ticks, and during eight months in the OeG ticks. This observation documents a potential mechanism for ASFV maintenance, as reservoir, for several months by Palearctic soft tick species (*O*. *erraticus* and *O*. *verrucosus*).

ASFV transmission by *O*. *erraticus* infected with Georgia2007/1 failed regardless of the number of ticks used per challenge (15 to 140), the number of challenges (1 or 3), or the time elapsed between tick infection and the transmission attempt (2 and 8 months). Although these findings are in agreement with previous observations by our research group [51], *O*. *erraticus* also failed to transmit the ASFV OURT88/1 strain included in our study design as a positive control. This strain was originally isolated from *O*. *erraticus* collected in Ourique, the Alentejo region of Portugal. Previous research documented that *O*. *erraticus* from this geographic area were able to transmit the virus to pigs [42]. Therefore, it will be important to confirm if relevant genetic differences between ASFV strains emerging in Eurasia are determinants of vector competence for *O*. *erraticus*, *O*. *verrucosus*, and other Palearctic soft tick species [52].

Previous studies also showed that vector competence can vary according to the ASFV strain and the soft tick populations tested where a gut barrier for generalization of infection before virus transmission occurs may exist [20]. However, the transmission failure in the OeG and OeO pairs is unlikely due to a tick gut barrier for infection. Productive infection, but not transmission, appears evident by the recovery infectious ASFV at time points later than 4 weeks PI, as shown by the outcome of the experiments where pigs were inoculated with tick homogenate [25, 27].The first experimental data testing the vector competence of *O*. *verrucosus* for ASFV are presented here. The scarce availability of *O*. *verrucosus* ticks limited the conduct of transmission trials with 30 OvZ ticks per pig, which yielded negative results. This outcome was similar to that observed with *O*. *erraticus*, with differences in ASFV loads at the 2-month PI time point as noted by the lower Ct values for OvZ. *O*. *verrucosus* is relatively small as compared to *O*. *moubata* and *O*. *erraticus*. Ukraine is considered the northern boundary of the geographical range for *O*. *verrucosus* [31], and its distribution appears to be associated with ecological niches such as riverine cliffs and limestone outcrops, which would limit its potential to be involved in the circulation of ASFV even if it had been shown to be a competent vector on these studies [53]. However, the results obtained with OvZ documented the ASFV reservoir ability of orally infected *O*. *verrucosus* for at least 2 months. Continued ecological changes in Ukraine promoting contact between *O*. *verrucosus* and susceptible hosts, e.g. through synanthropization, could results in the adaptation of ASFV strains to circulate in this native soft tick species.

Future studies will help to determine if differences in barriers other than the gut or genetic factors influence the relative vector competence for ASFV between populations of Palearctic soft tick species including *O*. *erraticus* [25, 52]. It will be important to determine if relevant genetic differences between closely related tick-transmissible and refractory strains exist, which could help identify potential vector competence determinants for ASFV.

Contamination of skin lesions with coxal fluid excreted during tick feeding is a possible mode for saliva-independent transmission as suggested by the ASFV titers in soft ticks and virus detection by PCR in washings obtained from mosquito netting retrieved after the attempted transmission in all the soft tick-virus pairs tested [15, 54]. Relevant differences in blood feeding biology exist between the ticks tested in our experiments. *O*. *moubata* excretes coxal fluid during the feeding process and after host detachment whereas coxal fluid excretion by *O*. *erraticus* and *O*. *verrucosus* occurs within several hours after feeding is completed [55]. We hypothesize that physiological variation in coxal fluid excretion could influence differences in vector competence for ASFV between *Ornithodoros* species. More investigations on viral titers in the ticks and their excreta, and visualization of ASV in tick tissues could help to understand the vector incompetence of *O*. *erraticus* in our experiments.

Our results verified the vector competence and the reservoir ability of *O*. *moubata* for Liv13/33. Horizontal transmission to pigs was successful at 2 and 8 months PI. This confirmed results from the study by Rennie [18], which detected ASFV 9 weeks PI in salivary glands of *O*. *moubata* artificially infected with Liv13/33. Vertical transmission to progeny was also observed, as previously reported [19]. Here, we demonstrated that infected progeny of female OmL ticks can transmit ASFV to pigs during their first blood meal.

Considering the presumed origin of the Georgia2007/1 strain [3], and the apparently widespread natural infection with ASFV genotype II in ticks from Southeast Africa [10], we hypothesized that *O*. *moubata* would successfully transmit this strain. OmG ticks were able to transmit ASFV at 2 and 6 months PI in single challenges using 30 ticks per pig. These results contrast findings in a previous report where a single infected soft tick *O*. *porcinus* was able to transmit ASFV to a susceptible pig [15]. Additional studies are needed to determine if the observed discrepancies are due to insufficient amounts of virus being transmitted under those challenge conditions [56, 57], or if the particular tick/virus strain combination influences vector competence [18, 21, 55].

It is critical to apply integrative taxonomy to the study of ticks and tick-borne diseases, including investigations on aspects related to vector competence. In the case of this study, the classical Afrotropical tick vector of ASFV is known by various names such as *O*. *porcinus*, *O*. *moubata porcinus* or simply as *O*. *moubata*. However, the most recent integrative taxonomic revision involving genetic and morphological analyses in the *O*. *moubata* group of ticks revealed that at least 4 distinct species (*O*. *moubata*, *O*. *phacochoerus*, *O*. *porcinus* and *O*. *waterbergensis*) could be potentially implicated in the transmission of ASFV with more species to be described if extensive geographical sampling were to be conducted [58]. At this point, an issue is to relate these new taxonomic data with previous studies on vector competence for ASFV in soft ticks. Looking forward, it is evident that work remains to be done to enhance our understanding of vector competence for ASFV given the diversity of soft ticks. This situation may also apply to other soft ticks as potential ASFV vectors that may include cryptic species like *O*. *erraticus* [25, 59] and *O*. *verrucosus* [33]. Sequencing and depositing or referencing voucher sequences of genetic markers commonly used in tick taxonomy should be a priority for vector competence studies [60], which will enhance our understanding of the role soft ticks can play as vectors of ASFV.

The genetic background of tick populations, their physiological status, or infections with other pathogens/endosymbionts, have been shown to significantly influence the competence of ticks as vectors of various infectious agents [60–62]. Therefore, predicting vector competence of a given tick species for ASFV will remain challenging, as long as the intrinsic determinants for successful tick infection and tick-borne transmission of ASFV remain to be fully determined. Future work involving comparative–omics approaches will offer the opportunity to explore the molecular basis of *Ornithodoros*-ASFV associations, as some viral genes apparently play an important role in infection and replication processes of ASFV in soft ticks [27, 63].

Finding a tick naturally infected with ASFV is not sufficient to predict its vector competence, which includes both the ability of the tick to become infected and then its ability to transmit to a susceptible animal [64]. Moreover, such a discovery does not predict the tick’s role in the epidemiology of ASF. A strict set of criteria to incriminate and define an arthropod as a vector or reservoir for a given pathogen exists in biomedical research [65, 66], with competence being only a part of the broader definition of vector capacity that in the case of arboviruses also includes extrinsic and intrinsic factors influencing their replication and transmission in nature by the biological vector. In view of the global ASF crisis, this study highlights the need to address the challenges related to the taxonomy, ecology and geographic distribution of *Ornithodoros* ticks in parts of the world where ASFV is emerging and re-emerging [31, 60].

## Supporting information

S1 Fig Clinical scores of pigs positive for ASFV that were infected in three different ways. Results are presented by boxplots. Cross correspond to the mean and horizontal lines correspond to the median.
(TIF)
