# Supplementary figures and images for: Comparative vector competence of the Afrotropical soft tick Ornithodoros moubata and Palearctic species, O. erraticus and O. verrucosus, for African swine fever virus strains circulating in Eurasia
Source: PLoS One. 2019 Nov 27;14(11):e0225657. doi: 10.1371/journal.pone.0225657 (PMC6881060; doi:10.1371/journal.pone.0225657)

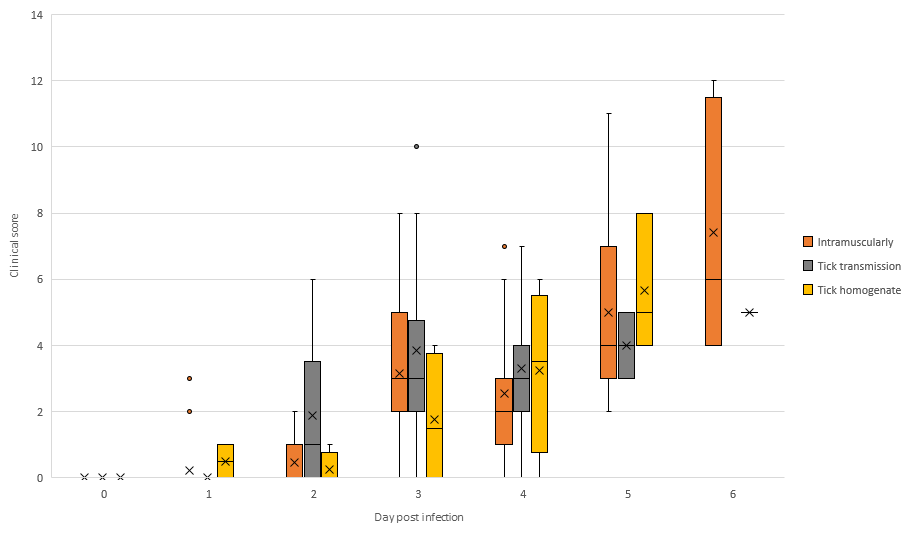

Supplement: S1 Fig — Results are presented by boxplots. Cross correspond to the mean and horizontal lines correspond to the median. (TIF) [file pone.0225657.s001.tif]
